# The Use of Haloperidol as a Sedative During Childbirth: An Extreme Form of Obstetric Violence in Spain

**DOI:** 10.3390/ijerph22010003

**Published:** 2024-12-24

**Authors:** Ibone Olza, Oscar Quintela, Araceli García-Martínez

**Affiliations:** 1 European Institute of Perinatal Mental Health, Association El Parto es Nuestro (Birth is Ours), 11406 Jerez de la Frontera, Spain; iboneolza@eipmh.com; 2 Department of Legal Medicine, Psychiatry and Pathology, Complutense University of Madrid, 28043 Madrid, Spain; oquintel@ucm.es

**Keywords:** maternal, pregnancy, reproductive rights, interventions, health policy, haloperidol, obstetric violence, chemical submission

## Abstract

Obstetric violence during pregnancy and childbirth is unfortunately a major problem throughout the world. Neuroleptanalgesia is a classic form of analgesia which consists in administering analgesics and neuroleptics, such as haloperidol, simultaneously. Haloperidol is still occasionally used during childbirth and, in most cases, without informed consent in Spain. It is used with the excuse of being an antiemetic, but the reality is that it is a form of obstetric violence called chemical submission. The combination of haloperidol with opioids leads to a potentiation of the sedative effects of both drugs, which may lead to multiplied risks for both mother and baby. At present, the use of haloperidol during childbirth is a practice exclusive to Spain. In fact, the association El Parto es Nuestro (Birth Is Ours) launched an awareness campaign in February 2021 aimed at eradicating the use of haloperidol during childbirth without informed consent. The present essay aims to bring awareness about the ongoing practice of using haloperidol. It is of great importance to eradicate this practice that is so harmful to mothers and their babies, as well as educate health personnel regarding this situation.

## 1. Introduction

Obstetric violence (OV) refers to a situation of mistreatment, disrespect, physical abuse, neglected care, verbal abuse, and non-consented care that women experience during pregnancy and labor and this alarming situation is prominent all over the world [1]. OV is a public health concern due to its prevalence rate in maternity care routines involving cultural, systemic, structural, and political-economic factors [1,2].

One form of OV is chemical submission: the unnecessary administration of medication with psychoactive effects to women during labor without informed consent. Recently, an editorial about chemical restraints for OV emphasized the fact that forced chemical compliance, or chemical submission, with unconsented obstetric interventions adds additional trauma to patients, contributing to short- and long-term patient harm. Considering that up to 44% of all women have traumatic experiences of their birth processes, it is necessary to become aware and review protocols, and healthcare professionals must embrace their role in protecting women from OV including forced chemical compliance [3].

In Spain, a specific form of OV consists in administering haloperidol to women during labor. The Association El Parto es Nuestro (Birth is Ours), a non-profit and feminist association that aims to improve the conditions of care for mothers and children during pregnancy, childbirth and postpartum in Spain [4] launched an awareness campaign (“Haloperidol during childbirth, never ever again”) in February 2021. This campaign specifically aims (1) to eradicate the administration of any drug during childbirth without informed consent; (2) to report the use of haloperidol during childbirth in Spanish hospitals; (3) to spread and raise awareness about the problem involved in the use of haloperidol during childbirth and why it must be eradicated; and (4) to spread the concept of chemical submission in childbirth. The present work describes the problem and presents the results of the campaign.

OV, including the use of haloperidol during childbirth, represents a profound violation of sexual and reproductive health and rights, as well as broader human rights. Framing this issue within these frameworks underscores its significance as not only a medical concern but also a matter of justice and equity. Recognizing OV as a violation of these fundamental rights highlights the urgent need for systemic change to ensure respectful, evidence-based, and rights-centered care during childbirth.

## 2. The Campaign to Eradicate the Use of Haloperidol

In this context, the Association El Parto es Nuestro (Birth is Ours) throughout the campaign (“Haloperidol during childbirth, never ever again”) launched a series of actions to denounce and eradicate this practice. Among other actions carried out, an open letter has been addressed to the Spanish Agency for Medicines, the Ministry of Health, and the main scientific associations in childbirth care requesting the eradication of the use of haloperidol as a sedative during childbirth.

In this letter, the association requests the following: a ban on haloperidol uses during labor; public communication to healthcare professionals on its risks and lack of justification; removal of this practice from midwifery training programs; research into its effects on mothers and newborns and support for affected women.

However, both institutions responded to the letter indicating that the matter did not fall within their scope of responsibility. The Association has written again to the Ministry of Health and the Mental Health Commissioner, urging them to act on this matter, but we have yet to receive a response.

We received many testimonies, mostly from midwives; some required anonymous treatment. It has come to our attention that haloperidol is still used in many public and private hospitals (we have detected at least 17 hospitals where haloperidol is still being used in Spain). Although the 2010 Clinical Practice Guidelines from the Strategy for Normal Birth Care (EAPN) [5] indicate meperidine as a possible opioid drug for analgesia in early labor and recommend the addition of an antiemetic, they do not detail which one or make any reference to haloperidol. However, we found, for instance, a study carried out at the Gregorio Marañón Hospital by midwife Fernández Arranz in 2019 [6] where she compared the use of the birthing ball with the pethidine and haloperidol cocktail, which proved it was still being used. Another study from 2022 described that at least 16 of 79 children were exposed to the lithic cocktail intrapartum in the University Hospital of Cartagena [7].

On the other hand, at present, protocols recommending haloperidol are still in force in many hospitals, but it is difficult to find this information: for instance, in the midwifery training guide of the Ministry of Health [8].

Some women know that they have been given Dolantine to relieve the pain of contractions but are unaware that it was administered together with haloperidol. This does not appear often in the discharge report. In our clinical practice, we have found that women who received haloperidol during labor describe experiences of disconnection, not being able to move or feeling drugged, with anguish and traumatic experiences during childbirth specially when the cocktail was administered to them without informed consent. Moreover, midwives describe the use of the cocktail to sedate non-cooperative women.

Regarding the physical and emotional experience of childbirth by mothers, we have collected at the Association El Parto es Nuestro (Birth is Ours) many testimonies of women who have had frightening birth experiences because of these drugs. For instance, many women describe feeling totally disconnected from their bodies, they have little or no recollection of what happened during the six hours or more following the injection. Others describe the distressing sensation of not being able to control their body movements. Among the different testimonies collected thanks to the campaign (through social media, Instagram, Facebook, Twitter, YouTube and email), the following are some examples:

*“Another nurse told me I wasn’t in labor yet but that she could give me something to help me. As soon as they added it to the drip, I lost consciousness. My partner realised that I was unable to talk. He looked at the drip and recognized the abbreviation for haloperidol (due to his occupation he is familiar with certain terms). By the time the drip had finished, I couldn’t stand on my two feet. I couldn’t string words together, it was as though I was literally drunk. As a matter of fact, I can’t recall anything that happened after that. My partner told me he left me slumped on the bed to go and look for a nurse; he wanted to know why I had been administered a psychiatric medication together with an opiate. And he wanted to know why we weren’t informed that such a medication was going to be administered, its consequences, etc. Different colleagues started to cover up for each other and finally one of them said that the only mistake was to have left the drip bag with its name to be seen, meaning they thought it best to use drip bags without indication of contents. We told them we wanted the medical report to state the fact they administered this medication to me, but they refused and to this day there is no record of it anywhere”.*

*“They tell me that I’m not dilating fast enough and that I’m going to be put on a drip with oxytocin. This made the pain increase considerably, of course, and I decided to drive it away with laboring cries (I’m an actress). It looks like they didn’t like that, and they told me they would put me on another drip with something to “calm me down”. I have no idea at all what it was that they called the mix, but from that point onwards I just lost complete control of myself and of what was happening. In the end, in the laboring room and during the expulsion stage I’d fall completely unconscious and either the contraction pains would suddenly make me come to my senses or they would slap me to wake me up, ordering me to push. I recall that during one of those “I thought I was dying. Everything ended with a Kristeller maneuver, an episiotomy they did not even previously warn me about and a ventouse on the head of my baby. I don’t know if “the mix” had haloperidol. All I know is that I have never ever been so unconscious not even after some of my worst drunken experiences during my youth”.*

*“They told me they could give me a painkiller sedative. I felt completely drugged, wishing the effect of whatever they had given me would go away. Later, talking to a midwife who attended me during the final stages, she told me the jab probably included haloperidol, which is what they use as an antiemetic, but before then no one had informed me of that at all. They said they were going to give me something for the pain; they didn’t say anything about nausea. Although, in any case, I didn’t have any. The pain didn’t go away, that’s for sure, despite the medication they gave me. I can describe the sensation perfectly. It was like being in a rowing boat in the middle of a storm. It was, of course, impossible to stay in any other position that wasn’t lying down. This together with the dizziness was stopping me from complaining about the pain which was the same as before said medication had been administered. It was an unforgettable experience”.*

In brief, not only is it necessary to take into account that these drugs are able to affect negatively and dangerously the baby and the mother, but also, this type of OV in the form of chemical submission may influence dramatically the dyad later, as has been shown in different studies on the relationship, for example, between OV and the development of postpartum depression and/or post-traumatic stress [9] or even the impact of this violence on quality of life and menopause-related disorders [10].

## 3. Discussion

### 3.1. The Use of Haloperidol During Childbirth

The use of a combination of opioids and neuroleptics dates to the 1950s, coinciding with the discovery of the first neuroleptics: chlorpromazine in 1952 and haloperidol in 1958 by Paul Janssen. For decades, both drugs became the first line of treatment for schizophrenia due to their blocking effect on dopamine D2 receptors [11]. Initially, the so-called “lithic cocktail” of Laborit and Huguenard was used: a combination of chlorpromazine (antipsychotic) with promethazine (antihistamine) and meperidine (opioid), where they observed its efficiency in rapidly treating the agitation of patients during the manic phase [12]. In 1959, two Belgian anesthesiologists, J. De Castro and P. Mundeleer, baptized this technique as neuroleptanalgesia (neuroleptics and analgesia), where they combined a major neuroleptic tranquilizer, usually droperidol or chlorpromazine, with a potent opiate narcotic analgesic, such as fentanyl or pethidine, thus achieving sedation, analgesia, and psychic indifference without loss of consciousness [13].

The first reference of use of the “lithic cocktail” during labor was found in 1956, when chlorpromazine was administered together with pethidine and promethazine to women who had preeclampsia or eclampsia to prevent and/or minimize the movement they had when convulsing and to also prevent shock [14]. At the time, when there was no effective treatment for eclampsia, it is understandable that a neuroleptic was given to prevent seizure movement. A 1959 paper on the advisability of giving prochlorperazine during labor mentioned “the attractive properties of tranquilizers regarding the obstetric patient” [15]. This use during labor was abandoned for a much more effective treatment for eclampsia: magnesium sulfate. In the sixties and seventies, several works describing the use of the “lithic cocktail” directly for the treatment of labor pain were published in Italy [16], France [17], Russia [18], Ukraine [19], and New Zealand [20]. These documents pointed out the convenience of giving an opioid to alleviate labor pain accompanied by the neuroleptic that produced stillness and indifference.

For instance, a paper by Staples in 1967 [21] evaluated the use of haloperidol together with meperidine in women during labor. This study included 50 women in active labor; 27 were injected intramuscularly with haloperidol and 23 with promethazine along with meperidine in both groups (‘or’ in conjunction, in both groups, with meperidine). Among the most notable results, they observed that both drugs (haloperidol and promethazine) exhibited a marked antiemetic effect; haloperidol potentiated the action of meperidine; no cases of depression in the newborn were found associated to the use of either drug (based on Apgar test) and there were no noticeable effects attributable to either drug on the post-partum course. Considering these results, it is not surprising that this “lithic cocktail” began.

A decade later, research published in Mexico in 1974 pointed out the possible advantages of associating droperidol with meperidine. The authors literally stated that “major tranquilizers produce psychic and motor sedation, with neurovegetative dampening which translates into psychic indifference and tranquility; in other words, emotional neutrality” [22]. They highlighted—as an advantage—that the use of droperidol during labor required a lower dose of meperidine, implying a possible benefit for the baby, and—as a disadvantage—a higher likelihood of requiring the use of forceps [22].

The introduction and subsequent generalization of epidural anesthesia as the main pharmacological relief for labor pain from the 1980s onwards meant that the “lithic cocktail” ceased to be in use [23]. Indeed, in 2010 a Cochrane review stated: “the use of lithic cocktail should be discontinued” [24]. In many places, e.g., the UK, Dolantine continued to be used for prodromal pain, together with drugs for nausea, such as metoclopramide or promethazine.

However, in Spain the practice of giving Dolantine and haloperidol for prodromal labor persists to this date without there appearing to be awareness regarding the risks of the cocktail or lithic mixture. When midwives are asked about this practice, many describe how midwives themselves indicate it, convinced that it is a safe practice and that “haloperidol is administered together with Dolantine to prevent or treat the nausea caused by Dolantine” [25]. This defense of haloperidol as an antiemetic is frankly astounding. In fact, according to Cochrane reviews [26,27], the use of haloperidol as an antiemetic should be reserved for patients in whom no other antiemetic has been effective. Nowhere is haloperidol indicated as a prophylactic antiemetic during labor, neither in Cochrane nor in any clinical studies.

### 3.2. The Problem with the Lithic Cocktail

Therefore, neuroleptics during labor were not introduced to treat nausea, but to enhance the sedation produced by opiates and especially to achieve this restraining effect and psychic indifference. Haloperidol has been specifically approved to treat schizophrenia, tics, and vocal utterances associated with Tourrette’s syndrome, and to treat hyperactivity and behavioral disorders, and only exceptionally is it used off-label for nausea and vomiting due to chemotherapy, advanced or terminal illness, and surgery. So, the original inclusion of haloperidol (or other neuroleptics) in the cocktail was never to use it as an antiemetic as is now being argued [28]. Moreover, its safety in the context of childbirth has never ever been investigated since the mentioned study in 1967, and it is important to remember that it can also reach the brain of the unborn child. Among the relatively frequent side effects of haloperidol are acute dystonia or involuntary muscle torsions or twitching that are very uncomfortable and sometimes painful. In a published case of a woman in labor who received the lithic cocktail in Spain, dystonia began during labor with oculogyric crises, which could only be resolved by administering biperiden once the baby was born [29]. Neuroleptics and prochlorperazine can also produce effects such as acute akathisia days after administration, so it is recommended not to use them as antiemetics in emergencies [29].

On the other hand, Dolantine, meperidine or pethidine, is a narcotic opioid which is not without risks [30]; it belongs to the same family as morphine which relieves pain quickly by acting directly on the brain. The main risk of this drug in the mother is that it can produce deep sedation and depression of the respiratory system. Moreover, as opioids cross the placenta, it may lead to reduced fetal heart rate variability, reduced baseline fetal heart rate, neonatal respiratory depression, lower Apgar scores, neurobehavioral alterations, and decreased early breastfeeding [30]. It is therefore considered a dangerous drug, requiring close monitoring. To all this, we must add that the combination of Dolantine and haloperidol has a synergistic or potentiating effect that transforms the cocktail into a powerful sedative, which on the one hand has an analgesic effect of pain relief, but also has the added effect of sedation.

Indeed, the supposed advantage of administering neuroleptics during childbirth was the chemical submission these drugs produced in women, which undoubtedly allowed other interventions to be performed during childbirth without consent and/or knowledge. In other words, the use of haloperidol during childbirth is not scientifically justified and, when given without true informed consent, it can be considered yet another example of OV in the form of chemical submission [31]. In this way, the situation described can be likened to the phenomena of chemical restraint used in other areas of medicine, such as geriatric care. Mothers in labor rooms become a vulnerable population that must be treated with respect and their consent for medical procedures that may arise during labor hours must be particularly scrupulously sought.

### 3.3. Preclinical Evidence of Prenatal Exposition to Haloperidol

A recent review about the prenatal exposition to haloperidol in animal models was published [32]. First-generation antipsychotic haloperidol can cross the placental tissue [33], but there is still very limited evidence regarding the outcomes in humans to the exposure to this drug during development. The safety of this drug in pregnant women and children is not yet established [32] and more studies are necessary.

It is important to also consider that the clinical effect of haloperidol is mediated by Cytochrome P450 (CYP). Genetic variations have allowed individuals to be classified as poor metabolizers (PM), intermediate metabolizers (IM), extensive metabolizers (EM), and ultra-rapid metabolizers (UM). For instance, PM usually suffer more adverse reactions at a normal dose of drug [34,35] (specifically, it is necessary to consider the CYP2D6 metabolism categories to determine the doses of haloperidol [36]). The severity of the response and side effects of haloperidol during labor is therefore highly variable depending on the genetic variations.

Nevertheless, there is growing evidence in animal models that prenatal exposure to haloperidol can severely affect brain development, both at molecular and cellular levels and at the behavioral level. For instance, at molecular and cellular levels prenatal haloperidol exposure has been found to affect the expression of plasticity-related genes (down-regulation of calmodulin, *N-CAM*, *GAP-43*, *PDGF-A* and *PDGF-A* receptor) [37], neurochemical-related genes (down-regulation of preproenkephalin and glutamic acid decarboxylase) [37], synapse-related and neuron-related genes (both up-regulated and down-regulated) [38], neurotrophic-related molecules, neurotransmitter system markers (including the dopaminergic system, the striatal peptidergic system, the cholinergic system, the serotoninergic system, and the glutamatergic and GABaergic system), and growth factors [32].

On the other hand, histological analysis has revealed region-specific morpho-cellular alterations due to the prenatal exposure to haloperidol, including the striatum [37,39]. Moreover, it was observed that haloperidol can impair the dendritic spine plasticity of the suprapyramidal and infrapyramidal dentate gyrus cells since it can significantly decrease the length of dendrites and the density of dendritic spines [34]. Reduced body and brain weight have also been described in rodents prenatally exposed to haloperidol from birth to youth and adulthood [40].

Taken together, these results suggest a negative impact on the offspring’s neurological development with short- and long-term consequences [32]. It is important to take into account that prenatal exposure early in development may be very different from prenatal exposure around the immediate time of delivery since there is some heterogeneity regarding the timing of when the prenatal exposure was in these animal models. The plasticity in different regions such as the prefrontal, hippocampus, amygdala complex, stria terminalis, and striatum is still present during the postnatal period; therefore, it could be very possible that the administration of haloperidol during labor has effects at molecular, epigenetic, and cellular levels probably appearing late [32]. Since the scientific and clinical relevance of the effects of haloperidol on neurodevelopment and the postnatal life of exposed fetuses is an undeniable fact, how is it possible that today it is a drug used during childbirth for sedative and antiemetic purposes when there are other, less aggressive alternatives? And what is worse, how is it possible that it is administered most of the time without the woman’s consent?

### 3.4. Difficulties and Limitations of the Campaign

Accessing information about hospital protocols regarding the use of haloperidol proved challenging, as such protocols are not publicly available, and the healthcare personnel involved were unwilling to grant access. Furthermore, in many cases, the use of haloperidol during labor, as described by the testimonies of surveyed women, was not documented in their medical records. Qualitative and quantitative studies need to be carried out to know the dimension and severity of the problem, as well as the impact on the health, both physical and mental, of mothers and babies in the short, medium, and long term. The Association El Parto es Nuestro (Birth is Ours) will continue gathering information and testimonies regarding this form of obstetric violence, aiming to raise awareness about the practice through papers, conference presentations, social media publications, and other communication channels.

## 4. Conclusions

Nowadays in Spain, women are still administered haloperidol during labor, a serious example of OV in the form of chemical submission. The Association El Parto es Nuestro (Birth is Ours) has been condemning this practice for years now. However, we continue to verify, with great concern, that this unsafe “cocktail” can still be found in the Guidelines for Normal Childbirth in many state hospitals. We have been gathering testimonies of mothers who felt drugged or absolutely knocked out, having lost complete control over themselves after receiving a “painkiller” at the beginning of their labors. In some cases, the consequences have been devastating.

The use of haloperidol during childbirth, as part of OV, constitutes a serious breach of sexual and reproductive health and rights, as well as fundamental human rights. Viewing this issue through these lenses emphasizes its importance beyond medical practice, framing it as a critical matter of justice and fairness. Acknowledging OV as a violation of these core rights highlights the pressing need for systemic reforms to promote care during childbirth that is respectful, evidence-based, and grounded in human rights principles.

The present work and the campaign developed by the Association El Parto es Nuestro (Birth is Ours), “Haloperidol during childbirth, never ever again”, aim to eradicate the use of haloperidol in childbirth. Moreover, it is of great relevance that the professionals involved in childbirth assistance know the pharmaceutical drugs that they are offering to women, and, above all else, always respect their informed consent.

## 5. Future Directions

The main objective of the article is to notify, denounce, and describe the existence and persistence of this practice considered OV. Through the campaign it has been possible to gather testimonies, two court rulings and information on the protocols of some hospitals. However, no research has been carried out as such. We suggest performing comparative case studies from each hospital and focused group discussions to find the best solution for the healthcare provider and the patients involved. We hope that this work serves as a framework or reference for the urgent development of an investigation with the aim of definitively eradicating this practice in Spain. By association, our work focuses on exposing and raising awareness about this type of obstetric violence. However, we believe it is the healthcare system and professionals who must take the appropriate measures to eradicate this practice.

## References

[1] Edward M.M., Kibanda Z. (2023). Obstetric violence: A public health concern. Health Sci. Rep..

[2] Sadler M., Santos M.J., Ruiz-Berdún D., Rojas G.L., Skoko E., Gillen P., Clausen J.A. (2016). Moving beyond disrespect and abuse: Addressing the structural dimensions of obstetric violence. Reprod. Health Matters.

[3] Burgart A., Sutton C. (2024). Chemical Restraints for Obstetric Violence: Anesthesiology Professionals, Moral Courage, and the Prevention of Forced and Coerced Surgeries. Am. J. Bioeth..

[4] Villarmea S., Olza I., Recio A. (2015). El parto es nuestro: El impacto de una asociación de usuarias en la reforma del sistema obstétrico de España. Dilemata.

[5] Ministry of Health and Social Policy and Equality Report on Attention to Delivery and Birth in the National Health System [Spanish]. https://www.mscbs.gob.es/organizacion/sns/planCalidadSNS/pdf/InformeFinalEAPN_revision8marzo2015.pdf.

[6] Fernández-Arranz J., Pedraz-Marcos A., Palmar-Santos A.M., Moro-Tejedor M.N. (2019). Birthing ball versus pethidine and haloperidol in satisfaction with childbirth. Enferm. Clin..

[7] Acosta E., Cortes O., Guzman S., Catala M., Lorente M., Arense J.J. (2022). Relationship between molar incisor hypomineralization, intrapartium medication and illnesses in the first year of life. Sci. Rep..

[8] Ministerio de Sanidad, Servicios Sociales e Igualdad (2014). Programa Formativo de la Especialidad de Enfermería Obstétrico Ginecológica (Matrona) Volumen 3. Enfermería Maternal y del Recién Nacido. Parte 3. Parto. Puerperio y Lactancia. Recién Nacido.

[9] Silva-Fernandez C.S., de la Calle M., Arribas S.M., Garrosa E., Ramiro-Cortijo D. (2023). Factors Associated with Obstetric Violence Implicated in the Development of Postpartum Depression and Post-Traumatic Stress Disorder: A Systematic Review. Nurs. Rep..

[10] Mendoza-Huertas L., Mendoza N., Godoy-Izquierdo D. (2024). Impact of violence against women on quality of life and menopause-related disorders. Maturitas.

[11] Tyler M.W., Zaldivar-Diez J., Haggarty S.J. (2017). Classics in Chemical Neuroscience: Haloperidol. ACS Chem. Neurosci..

[12] Kunz E. (2014). Henri laborit and the inhibition of action. Dialogues Clin. Neurosci..

[13] Castro D., Mundeleer P. (1959). Anesthesia without sleep: “neuroleptanalgesia”. Acta Chir. Belg..

[14] Hudson E.G., Siew S.C. (1956). The phenothiazine derivatives in the treatment of eclampsia; a preliminary report. J. Obstet. Gynaecol. Br. Emp..

[15] The Effect of Prochlorperazine on Uterine Contractions A Clinical and Experimental Study. https://pubmed.ncbi.nlm.nih.gov/13841400/.

[16] Neuroleptoanalgesia In Obstetrics. https://pubmed.ncbi.nlm.nih.gov/14240290/.

[17] Study of the Fetal and Maternal Effects of Neuroleptanalgesia by Biologic and Electronic Surveillance of Labor. https://pubmed.ncbi.nlm.nih.gov/4681577/.

[18] Neuroleptoanalgesia in Labor. https://pubmed.ncbi.nlm.nih.gov/4761896/.

[19] Anesthesia and Acceleration of Labor Using Neuroleptic and Analgesic Agents. https://pubmed.ncbi.nlm.nih.gov/4463328/.

[20] Neuroleptanalgesia in Labour. https://pubmed.ncbi.nlm.nih.gov/4524820/.

[21] The Use of Haldol (Haloperidol) in Obstetrics. https://pubmed.ncbi.nlm.nih.gov/20468140/.

[22] Dehydrobenzperidol and Its Effect on the Contractility of the Nonpregnant Human Uterus Preliminary Report. https://pubmed.ncbi.nlm.nih.gov/4149587/.

[23] Halliday L., Nelson S.M., Kearns R.J. (2022). Epidural analgesia in labor: A narrative review. Int. J. Gynaecol. Obstet..

[24] Duley L., Gülmezoglu A.M., Chou D. (2010). Magnesium sulphate versus lytic cocktail for eclampsia. Cochrane Database Syst. Rev..

[25] Roura L.C., Sociedad Española de Ginecología y Obstetricia SEGO (1996). Manual del Residente de Obstetricia y Ginecología (SEGO).

[26] Murray-Brown F., Dorman S. (2015). Haloperidol for the treatment of nausea and vomiting in palliative care patients. Cochrane Database Syst. Rev..

[27] Weibel S., Rücker G., Eberhart L.H.J., Pace N.L., Hartl H.M., Jordan O.L., Mayer D., Riemer M., Schaefer M.S., Raj D. (2020). Drugs for preventing postoperative nausea and vomiting in adults after general anaesthesia: A network meta-analysis. Cochrane Database Syst. Rev..

[28] Guia de Parto de Baja Intervencion. http://www.sspa.juntadeandalucia.es/servicioandaluzdesalud/hinmaculada/web/servicios/tcg/recursos%20y%20datos/Protocolos/Nuestro%20Protocolo%20Parto/GUIA%20DE%20PARTO%20DE%20BAJA%20INTERVENCION.pdf.

[29] Martínez Fernández G., Plaza Moral A., Miró Descarga P., Arguís Gimeno M.J., Gomar Sancho C. (2004). Acute dystonia due to haloperidol during labor. Rev. Esp. Anestesiol. Reanim..

[30] Smith L.A., Burns E., Cuthbert A. (2018). Parenteral opioids for maternal pain management in labour. Cochrane database Syst. Rev..

[31] Cruz-Landeira A., Quintela-Jorge Ó., López-Rivadulla M. (2008). Chemical submission, epidemiology and some clues for the diagnosis. Med. Clin..

[32] Santos A.V.S., Cardoso D.S., Takada S.H., Echeverry M.B. (2023). Prenatal exposition to haloperidol: A preclinical narrative review. Neurosci. Biobehav. Rev..

[33] Newport D.J., Calamaras M.R., DeVane C.L., Donovan J., Beach A.J., Winn S., Knight B.T., Gibson B.B., Viguera A.C., Owens M.J. (2007). Atypical antipsychotic administration during late pregnancy: Placental passage and obstetrical outcomes. Am. J. Psychiatry.

[34] Zhao M., Ma J., Li M., Zhang Y., Jiang B., Zhao X., Huai C., Shen L., Zhang N., He L. (2021). Cytochrome P450 Enzymes and Drug Metabolism in Humans. Int. J. Mol. Sci..

[35] Foster A., Mobley E., Wang Z. (2007). Complicated pain management in a CYP450 2D6 poor metabolizer. Pain Pract..

[36] Milosavljević F., Bukvić N., Pavlović Z., Miljević Č., Pešić V., Molden E., Ingelman-Sundberg M., Leucht S., Jukić M.M. (2021). Association of CYP2C19 and CYP2D6 Poor and Intermediate Metabolizer Status With Antidepressant and Antipsychotic Exposure: A Systematic Review and Meta-analysis. JAMA Psychiatry.

[37] Castro R., Brito B., Segovia J., Martin-Trujillo J.M., Notario V. (1994). Prenatal haloperidol induces a selective reduction in the expression of plasticity-related genes in neonate rat forebrain. Mol. Brain Res..

[38] Yoshino Y., Kumon H., Shimokawa T., Yano H., Ochi S., Funahashi Y., Iga J.I., Matsuda S., Tanaka J., Ueno S.I. (2022). Impact of Gestational Haloperidol Exposure on miR-137-3p and Nr3c1 mRNA Expression in Hippocampus of Offspring Mice. Int. J. Neuropsychopharmacol..

[39] Singh K.P., Singh M. (2002). Effect of prenatal haloperidol exposure on behavioral alterations in rats. Neurotoxicol. Teratol..

[40] Wang H., Li J.T., Zhang Y., Liu R., Wang X.D., Si T.M., Su Y.A. (2019). Prenatal Exposure to Antipsychotics Disrupts the Plasticity of Dentate Neurons and Memory in Adult Male Mice. Int. J. Neuropsychopharmacol..

